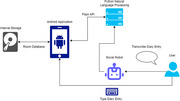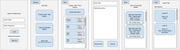# From Journaling to Insights: Empowering People with Dementia using AI and Social Robotics

**DOI:** 10.1002/alz70858_101504

**Published:** 2025-12-25

**Authors:** Toby Surtees, Aditya Purswani, Neil Chadborn, Armaghan Moemeni

**Affiliations:** ^1^ University of Nottingham, Nottingham, Nottinghamshire, United Kingdom

## Abstract

**Background:**

Dementia is a prevalent, global disease with a high carer burden. Despite its pervasive impact, effective treatment remains elusive due to the condition's intricate multi‐factorial nature. Traditional pharmacological treatments are costly and ineffective for many, necessitating exploration of alternative interventions. This study presents an application that aims to enhance the daily life of people living with dementia (PwD). Advances in Artificial Intelligence (AI), specifically with Natural Language Processing (NLP) and Social Robotics, bring opportunities to address this. This is particularly helpful for PwD who experience emotional and psychological changes, as well as language and cognitive impairments.

**Method:**

We have studied the impact of integrating AI and assistive living technologies in gerontology for older PwD. By integrating these technologies, this project serves as a key enabler for research in assistive technologies for dementia care, giving opportunities ‐ e.g. feedback from PPI ‐ to identify where support is most needed, providing better‐targeted interventions, and offering insights to inform the development of personalised support. Social robots are deployed to interact with humans; NLP used to analyse text to extract useful details. Accordingly, an interactive mobile app developed and integrated with MiRo© (robot) for companionship, capable of taking input diary entries from PwD, via voice or typing (Figure 1 & 2). A list of ’Insights’ then created and given to the PwD and registered caregivers to view for mood tracking, organizing events and tracking medications. Caregivers and family members can remotely view insights from their own devices, providing stress‐free, non‐intrusive monitoring of their loved ones.

**Result:**

A meeting has been organized with five members of the PPI (Patient and Public Involvement) to delve into specific themes and issues that had emerged while proposing this application. Furthermore, semi‐structured interviews and surveys are planned with PPI to assess usability and effectiveness of this app in everyday life of PwD (March 2025).

**Conclusion:**

Initial findings have guided specification designs and product development. From meetings with PPI, we will determine the effectiveness of this application in daily living of PwD, we will be able to specifically target improvements for the software to improve it using feedback from the target audience.